# Gaze-contingent display technology can help to reduce the ipsilesional attention bias in hemispatial neglect following stroke

**DOI:** 10.1186/s12984-022-01104-5

**Published:** 2022-11-16

**Authors:** Lisa Kunkel genannt Bode, Anna Sophie Schulte, Björn Hauptmann, Thomas F. Münte, Andreas Sprenger, Björn Machner

**Affiliations:** 1 Department of Neurology, University Hospitals Schleswig-Holstein, Campus Lübeck, Ratzeburger Allee 160, 23538 Lübeck, Germany; 2 Neurological Center Segeberger Kliniken, Bad Segeberg, Germany; 3 Center of Brain, Behavior and Metabolism, University of Lübeck, Lübeck, Germany; 4 Department of Psychology II, University of Lübeck, Lübeck, Germany; 5 Department Performance, Neuroscience, Therapy and Health, Medical School Hamburg, Hamburg, Germany

**Keywords:** Spatial neglect, Salience modification, Gaze-contingent displays, Attentional priority map

## Abstract

**Background:**

Hemispatial neglect results from unilateral brain damage and represents a disabling unawareness for objects in the hemispace opposite the brain lesion (contralesional). The patients’ attentional bias for ipsilesional hemispace represents a hallmark of neglect, which results from an imbalanced attentional priority map in the brain. The aim of this study was to investigate whether gaze-contingent display (GCD) technology, reducing the visual salience of objects in ipsilesional hemispace, is able to rebalance this map and increase awareness and exploration of objects in the neglected contralesional hemispace.

**Methods:**

Using remote eye-tracking, we recorded gaze positions in 19 patients with left hemispatial neglect following right-hemisphere stroke and 22 healthy control subjects, while they were watching static naturalistic scenes. There were two task conditions, free viewing (FV) or goal-directed visual search (VS), and four modification conditions including the unmodified original picture, a purely static modification and two differently strong modifications with an additional gaze-contingent mask (GC-LOW, GC-HIGH), that continuously reduced color saturation and contrast of objects in the right hemispace.

**Results:**

The patients’ median gaze position (Center of Fixation) in the original pictures was markedly deviated to the right in both tasks (FV: 6.8° ± 0.8; VS: 5.5° ± 0.7), reflecting the neglect-typical ipsilesional attention bias. GC modification significantly reduced this bias in FV (GC-HIGH: d = − 3.2 ± 0.4°; p < 0.001). Furthermore, in FV and VS, GC modification increased the likelihood to start visual exploration in the (neglected) left hemifield by about 20%. This alleviation of the ipsilesional fixation bias was not associated with an improvement in detecting left-side targets, in contrast, the GC mask even decreased and slowed the detection of right-side targets. Subjectively, patients found the intervention pleasant and most of the patients did not notice any modification.

**Conclusions:**

GCD technology can be used to positively influence visual exploration patterns in patients with hemispatial neglect. Despite an alleviation of the neglect-related ipsilesional fixation bias, a concomitant functional benefit (improved detection of contralesional targets) was not achieved. Future studies may investigate individualized GCD-based modifications as augmented reality applications during the activities of daily living.

**Supplementary Information:**

The online version contains supplementary material available at 10.1186/s12984-022-01104-5.

## Background

Hemispatial neglect represents a disabling cognitive disorder following unilateral, usually right-hemispheric stroke [1]. Affected patients are impaired in responding and spontaneously orienting to stimuli (objects, persons and even own body parts) in the contralesional hemispace [2]. ‘Contralesional’ thereby denominates the hemispace opposite the brain lesion, e.g., the left hemispace is ‘contralesional’ for right-hemispheric stroke patients. For these patients, the right hemispace is on the same side as the (right-hemispheric) brain lesion, i.e. it is ‘ipsilesional’. Neglect is an independent predictor of poor functional outcome following a stroke and the affected patients often need permanent care and require intense rehabilitation [3].

The core deficit of neglect is a bias in spatial attention towards the ipsilesional half of space within an egocentric reference frame centered on the observer (‘ipsilesional attention bias’) [1, 4]. The observation that patients with neglect can additionally (or even exclusively) have an unawareness for the contralesional part of an object independent of its position in space has been interpreted as a separate entity called object-centered or ‘allocentric’ neglect [5, 6]. However, some regard this object-centered neglect as a relative egocentric neglect, which would be further in line with the ipsilesional attention bias in egocentric space as the hallmark of hemispatial neglect [7, 8].

Whether attention is directed to a specific object in space depends on its (bottom-up) salience and its (top-down) relevance [9–11]. As the neural basis for this process of selective attention an “attentional priority map” in the brain has been proposed [12–15]. In patients with hemispatial neglect due to unilateral brain damage this map is assumed to be imbalanced, objects in ipsilesional hemispace are weighted as more salient/relevant, while contralesional objects underlie a permanent attentional disadvantage and are less likely to be attended [14, 16, 17].

Eye-tracking techniques can measure the distribution of fixations (gaze positions) in a visual scene, visualizing the overt attentional shifts to objects in space [18, 19]. Thereby they can uncover the ipsilesional attention bias in neglect patients during visual exploration, as their fixations are strongly shifted to the ipsilesional hemifield both in visual search (VS) [20] and in free viewing (FV) tasks [21–23].

Particularly in the very early phase of attentional orienting, e.g., during the first few seconds or the first fixations, patients with left hemispatial neglect exhibit a significant rightward bias in their visual exploration pattern [20, 24]. Likewise, this early ipsilesional orientation bias occurs during the execution of cancellation tasks, i.e., tasks in which several target objects on a sheet of paper have to be cancelled out with a pencil. Notably, the starting point (position of the first object cancelled on the paper) has been shown to be one of the most sensitive diagnostic tools for the detection of spatial neglect [25]. While healthy control subjects usually start the exploration/cancellation in the left hemifield, patients with left neglect typically start on the right [20, 26].

Via eye-tracking techniques it was previously shown that the fixations of patients with neglect are influenced by the objects’ salience, i.e. physical pop-out characteristics like contrast, color and motion, even if the objects are located in the neglected contralesional hemispace [27–29].

Gaze-contingent display (GCD) technology, manipulating the visual salience of objects in dependence of their position in space, could represent a promising tool to change the pathological visual exploration pattern of neglect patients. The basic principle of GCDs is that first the observer’s current gaze position (fixation) in a visual scene is recorded with a highly accurate eye tracker [30, 31]. Then, a computer-generated mask (e.g., inducing a peripheral or hemifield blur) is superimposed on the visual image centered on the current gaze position, and it is continuously updated with every new gaze jump (saccade). By application of fast programming algorithms, this masking happens with such a short latency (usually < 20 ms) that the observer is not perceiving the original image before positioning of the mask and also no ‘smearing’ of the manipulated image during visual exploration. In a previous study [11], our research group could show that GCDs were able to negatively influence visual exploration patterns of healthy subjects in both FV and goal-directed VS. Using different static and dynamic masks, which gradually reduced the salience of objects in the left hemifield, we induced a neglect-like visual exploration pattern in healthy subjects including a rightward shift of fixations in the early phase of orienting, avoidance of the far left of the visual scene and hyper-exploration of the far right.

The aim of the current study was to investigate whether the reverse modification, i.e. a GCD permanently reducing the visual salience of objects in the right (ipsilesional) hemispace, is able to externally counterbalance the internal tilt of the attentional priority map and direct the patients’ gaze to objects in the neglected (left) hemispace.

The current approach somewhat resembles the constraint-induced therapies that are used in the field of motor rehabilitation for stroke patients, i.e., the use of the intact ipsilateral limb is restricted in order to enhance the use and functional recovery of the paretic contralesional limb [32–34]. In stroke patients with neglect, such an approach has been previously tested by targeting the visual domain. Hemifield eye patches on glasses restricted the visual information from the intact ipsilesional hemifield and enhanced visual exploration and motor initiation towards the neglected contralesional hemispace, but its impact on functional recovery remained open [35, 36].

We applied two different task conditions, each investigating a different subtype of visual exploration: (i) FV that is solely driven by bottom-up information in the visual scene, i.e. the distribution and physical properties (e.g., color and contrast) of objects [37, 38] and (ii) goal-directed VS that is mainly controlled by top-down signals (e.g., task instruction) and less influenced by bottom-up information [39, 40]. Both tasks combined with eye movement recordings were previously shown to be feasible and reliable tools for diagnosing hemispatial neglect in stroke patients [20, 23, 41]. Kaufmann et al. [42] could further show a very good test–retest reliability and recommended the paradigm for treatment trials with repeated measurements. In the current study, we accordingly used eye movement recordings during FV and VS to not only induce gaze-dependent saliency modifications but also to ‘read out’ overt attentional shifts in order to assess the impact of the modifications on the visual exploration behavior of neglect patients. In this study, we specifically asked whether a bottom-up salience modification would positively influence the patients’ visual exploration pattern in both FV and VS and whether a potential normalization of the ipsilesional exploration bias would also go along with a functional benefit, i.e. an improved detection of targets in the neglected hemispace during VS.

## Methods

### Participants

Patients with left hemispatial neglect were recruited at the neurological rehabilitation center in Bad Segeberg, Germany. Inclusion criteria were (i) age of 18 years or older, (ii) right-handedness, (iii) a first-ever stroke (ischemic or hemorrhagic) in the right hemisphere and (iv) left hemispatial neglect as confirmed by a neuropsychological test battery consisting of Text Reading [43], Word reading [44], Bells Test [45], Line Bisection [46], and Figure copying [47]. Exclusion criteria were a neurodegenerative disease (e.g., dementia, Parkinson’s disease), structural lesions of the brain besides the stroke, low vision (corrected < 0.7) or an inability to give informed consent. For the control group, we recruited healthy subjects via announcements on social media platforms as well as on bulletin boards at the campus of the University of Lübeck. In the announcements we explicitly asked for participants older than 45 years in order to better match the expected mean age of the stroke patient group. Exclusion criteria were neurological, psychiatric, or ophthalmological diseases (actual or in the past) and left-handedness. To exclude a hitherto unrecognized disorder of spatial attention, all control participants underwent the Bells Cancellation and Line Bisection as screening tests. Control subjects were investigated at the Center of Brain, Behavior and Metabolism on the campus of the University of Lübeck.

Of 24 recruited patients with hemispatial neglect, five had to be excluded due to insufficient quality of the eye tracking signal, leaving 19 patients (9 women) for final analysis. Their mean age was 66 years (SD = 10) and the time since stroke was on average 105 days (SD = 124, range 15 to 530 days), which relates to a subacute or chronic stage of stroke. After exclusion of one healthy control participant due to poor eye tracking data, the control group consisted of 22 participants (15 women, 7 men), whose mean age was 55 years (SD = 10).

The patient and the control group differed significantly in age (*t*(39) = 3.773, *p* = 0.001), patients were on average 11 years older. There was no difference concerning the gender distribution (Mann Whitney *U* = − 1.332, *p* = 0.183).

The demographic, clinical and neuropsychological characteristics of the sample are shown in Table 1 in more detail. All participants were right-handed. For individual data of each study participant see Additional file 5: Tables S1 (patients) and S2 (healthy controls) in the supplement.

**Table 1:** Demographic, clinical and neuropsychological characteristics of the study sample

	Range	Patients (N = 19)	Control (N = 22)
Demographic			
Age in years (SD)		66 (10)	55 (10)
Sex (Female:Male)		9:10	15:7
Clinical			
Time since lesion in days (SD)		105 (124)	
Barthel Index (SD)		42 (9)	
Type of stroke (ischemic: hemorrhagic)		11:8	
Neuropsychological			
Text reading [43]: Words omitted or incorrect [n]	0 to 180	18.4 ± 6.5	
Word list reading [44]: Words incorrect [n]	0 to 40	3.7 ± 0.9	
Bells Test [45]:			
Targets missed	0 to 35	15.1 ± 2.6	0.3 ± 0.1
Center of Cancellation	− 1 to + 1	0.39 ± 0.08	0 ± 0
Line bisection [46]:			
Deviation from center [mm]	0 to 200	33.1 ± 6.6	0.2 ± 0.6
Figure copying (Ogden) [47]: Score	0 to 4	2.9 ± 0.3	
Clock drawing [49]: Score	0 to 5	3.8±0.3	

Data are reported as Mean (SD) or Mean ± SEM

### Eye tracking device and setup

Participants were seated in a dim lit room in front of a 23″ widescreen TFT monitor (Hanns-G HS233H3B, resolution of 1920 × 1080 pixels and a refresh rate of 120 Hz). At an eye-to-screen distance of 75 cm the display covered a visual field of 38° width and 22° height. Eye movements were recorded using a remote eye tracking device (EyeLink Portable Duo at 500 Hz or EyeLink 1000 Plus, SR Research Ltd, Ontario, CA, USA), which allowed contact-free eye tracking and head-free investigations due to an integrated head motion compensation.

The standard calibration procedure of the EyeLink-system with a 13-point calibration was used, including a manual acceptance of the calibration points. Since the reference frame for the static modification mask was the absolute center of the screen independent of the observer’s trunk and the dynamic mask for the GC-modifications was always centered on the participant’s current gaze position, there was no further need to control for the participant’s individual head or trunk position during the experiment.

### Gaze-contingent display technology

GCD technology was used to continuously modify the visual stimulus in dependence of the current gaze position. Therefore, we first derived the signal from the eye tracker to localize the current gaze position on the screen. Next, using MATLAB® with the parallel programming toolbox, the respective GC-mask (see modifications) was superimposed on to the original image, always centered on the current gaze position. Every update of the mask after a change of the gaze position following a gaze jump (saccade) took less than eight milliseconds, taking all steps and system delays into account (eye-tracking signal detection, mask programming, screen refresh). This rapid update prevented participants from perceiving the stimuli in their original salience before the mask covered the respective area on the picture.

### Task conditions and stimulus images

There were two different *task conditions*, (FV and VS, each having a different set of stimulus images.

In the FV task, the stimulus images were different photographs of landscapes, food, residential scenes, etc., which were taken from a freely available online stock (https://pixabay.com; for examples see Fig. 2). We chose 40 photographs with a rather homogenous content and symmetric left–right distribution of objects. However, the images were always presented twice, once in their original version and once in a mirrored (flipped) version to control for potential differences in the image’s salience along the x-axis and to exclude any stimulus-driven bias during the visual exploration. Each image was presented for twelve seconds. Participants were asked to attentively watch the photographs without any specific task given.

For the VS task, we used 80 different naturalistic images of a desk scene [48]. In each scene there were 30 different every-day objects (pen, key, etc.) scattered on a desk. Patients were instructed to search for the target, a paperclip that could be either red or blue. The image remained present until a response (spacebar-press) was made but only up to a maximum of twelve seconds. A target was present in 80% of the trials. Regarding its horizontal position on the screen, the target was located within one of four columns (outer left [OL], center left [CL], center right [CR], outer right [OR]) at an equal probability.

### Modifications

**Fig. 1:**
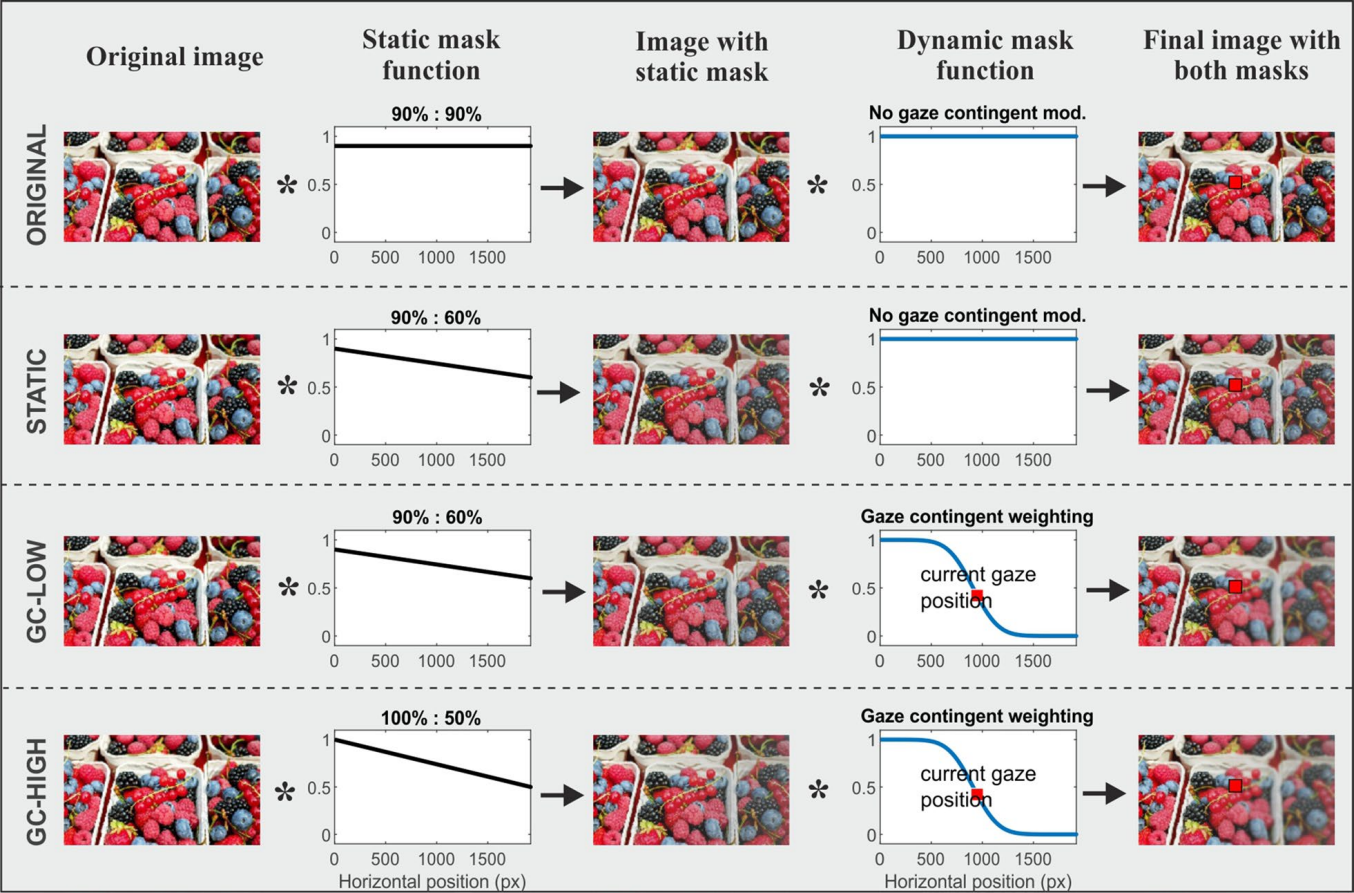
Modifications. An exemplary stimulus picture (“Berries”) is depicted in its original version at 90% color saturation (ORIGINAL) as well as with one of three modification masks, i.e. STATIC (90–60% left–right gradient of color saturation), GC-LOW (90–60% static gradient of color saturation + dynamic GC mask continuously reducing the contrast on the right of current gaze position), GC-HIGH (100–50% left–right gradient of color saturation + the dynamic GC mask)

Independent of the task condition, the stimulus image was presented either in its original version at a color saturation of 90% (ORIGINAL) or in one of three modified conditions, in which a mask superimposed onto the image reduced the salience of objects along a left–right gradient (Fig. 1). The masks were constructed based on the theory of an imbalanced attentional priority map in neglect patients, i.e., they reduced the visual salience of objects in the ipsilesional hemispace and thereby relatively increased the salience of stimuli in the contralesional hemispace. Hence, these masks were created to externally counterbalance the internally tilted attentional priority map.

In the STATIC modification, the mask was stable on the screen with regards to the viewer’s trunk position (“body-centered”) and constantly reduced the color saturation of objects in the image following a left–right-gradient from 90% at the left border to 60% at the far right. In the GC-LOW modification, this static “body-centered” mask was combined with a dynamic “eye-centered” mask that was permanently updated dependent on the current gaze position. This dynamic mask induced an additional “blur” by decreasing the local contrast of objects on the right of the current fixation. The dynamic mask was programmed using the”imgaussfilt” function within MATLAB® and applying the same parameters as in our previous study [11].

The strongest modification was the GC-HIGH, for which the gradient of the static mask was further increased (color saturation decreasing from 100% on the extreme left to 50% on the extreme right) and again combined with the dynamic GC mask described above.

The additional movie files 1—4 provide a visual impression of how the masks modified the stimulus images’ salience by showing original scanpaths of one exemplary patient under the four different modifications (Additional file 1 (ORIGINAL), Additional file 2 (STATIC), Additional file 3 (GC-LOW) and Additional file 4 (GC-HIGH)).

### Experimental procedure

**Fig. 2:**
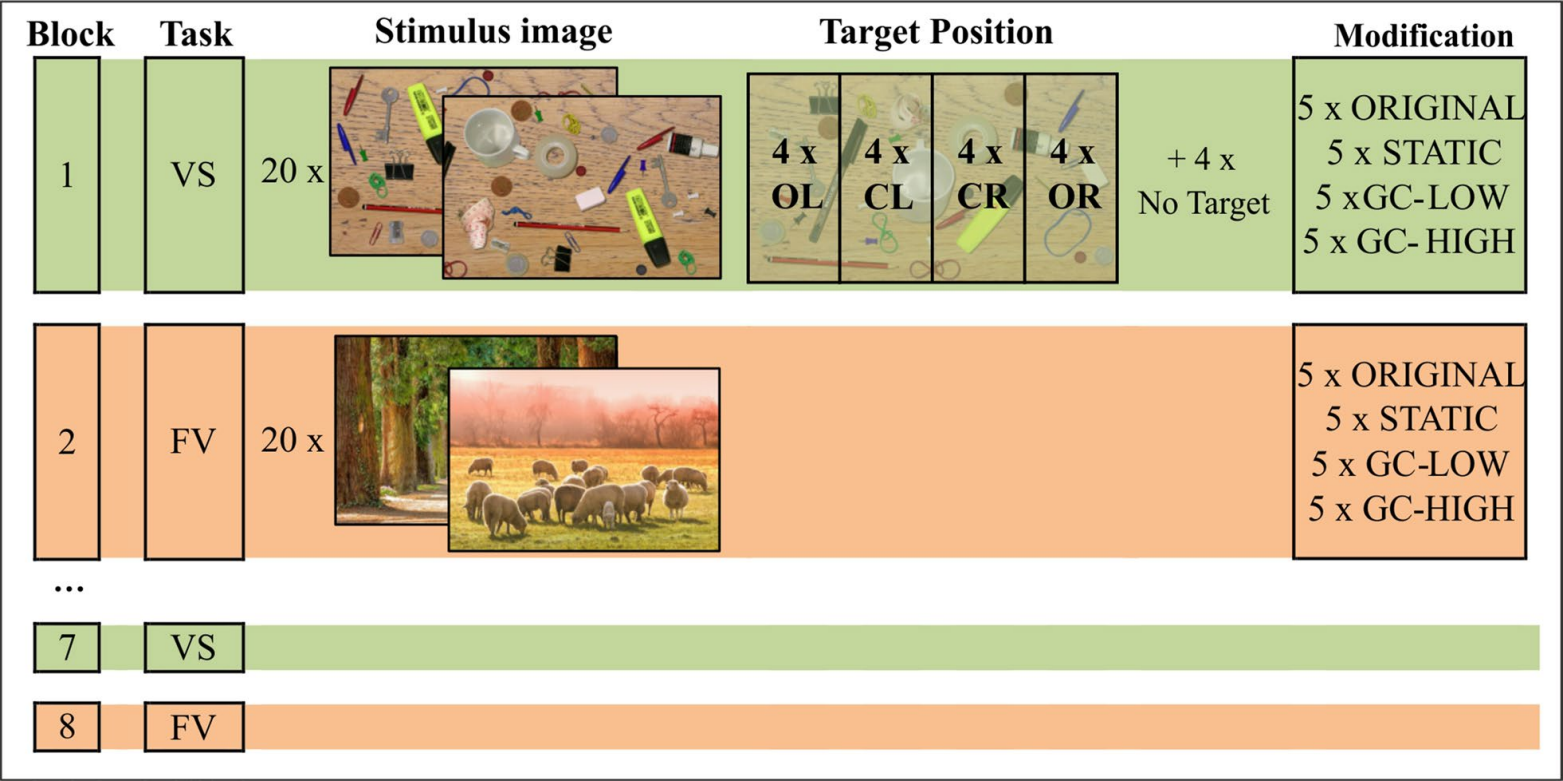
Trial design. Participants underwent alternating blocks of the Free Viewing (FV) and Visual Search (VS) task. Each block included 20 trials encompassing an equally balanced number (n = 5) of the four types of modification. In the VS task, the target could be present in one of four columns (outer left [OL], center left [CL], center right [CR], outer right [OR]) or it was missing, each with an equal probability

After positioning of the participant and calibration of the eye tracking device, the experiment always started with the first VS block followed by the first FV block (Fig. 2). Each block consisted of 20 trials, including an equal number of trials (n = 5) for all four types of modification, presented in a randomized order. There was a maximum of 8 blocks (4 blocks for each task), presented in a fixed alternating order of VS-FV-VS- and so on. Each trial lasted up to a maximum of twelve seconds, resulting in a total duration of the experiment of max. 32 min (excluding potential breaks).

### Outcome parameters

As a parameter of the spatial attention bias, we analyzed the *Center of Fixation* (CoF), defined as the median x-position [°] of all gaze points on the stimulus screen (range -19° to + 19°), i.e. 50% of all gaze points were located on the left and 50% on the right of this x-coordinate. As marker of the early orienting, we analyzed for each condition the percentage of trials that started with a leftward saccade (*First orienting [% leftward]*). As parameters of functional relevance in everyday life, in the VS task we analyzed the *omission rate,* defined as the proportion [%] of target-present trials in which the target was not detected, as well as the *reaction time* (RT), i.e. the time in seconds until the target response button was pressed in target-present trials. If there was no button press in a target-present trial, the RT was set at the maximum time of 12 s. As a parameter of feasibility and convenience of the experimental setup, aiming at a potential administration during neglect rehabilitation in the future, patients were asked after the experiment whether they perceived the tasks as pleasant or annoying (scale from 1 very annoying to 10 very pleasant) and whether they want to report any observations (in order to check whether the modification was noticed at all or if it was too strong).

### Statistics

Statistical analyses were performed using SPSS (version 22). Data are reported as Mean ± Standard Error of the Mean (SEM). The two task conditions (FV, VS) were analyzed separately. To assess the influence of the modifications on the different outcome parameters, we performed ANOVAs with repeated measures using GROUP as between-subject factor, MODIFICATION as one within-subject factor and in VS the COLUMN (target position) as another within-subject factor. If the sphericity requirement was violated, the F-values and p-values are reported with Greenhouse–Geisser correction, but degrees of freedom are reported uncorrected for clarity of the analysis design. In case of significant main effects, the relevant post-hoc two-tailed t-tests were performed and reported if they attained a statistical level of *p* < 0.05 (Bonferroni correction applied for multiple testing).

## Results

### Exclusion of trials after quality control of the eye movement data

In one neglect patient and one control subject there were technical problems during the presentation of the VS task and the associated recording of the eye movements, which led to the exclusion of all their VS trials from the analysis. Furthermore, single trials were excluded from analysis in all participants, if their number of valid data points (gaze samples on the screen/ presentation duration [ms]) was less than two standard deviations below the group mean. This applied to 5% of the trials in the FV and 4.6% in the VS blocks in the neglect patient group as well as to 6.5% of the trials in FV and 6.1% in VS in the control group.

### Outcomes in the FV task

All the control subjects completed the four FV blocks, the patients had less attentional and/or motivational capacities and finished on average 3 (SD = 0.8) blocks. The data of all the relevant outcome measures in the FV and the VS task are presented in Table 2. Furthermore, individual results of the patients are provided in the supplement (Additional file 5: Tables S3–S6).

**Table 2:** Overview of the results

		Neglect				Control			
		Original	Static	Gc-low	Gc-high	Original	Static	GC-low	GC-high
Free viewing									
Center of Fixation [°]		6.8 ± 0.8	6.2 ± 0.8	3.8 ± 0.9	3.6 ± 0.9	0.3 ± 0.2	0.4 ± 0.2	0.5 ± 0.4	0.2 ± 0.4
First orienting leftward [%]		13.6 ± 3.1	13.2 ± 3.4	23.3 ± 5.3	35.3 ± 6.7	44.6 ± 5.1	50.1 ± 4.6	70.5 ± 5.5	72.7 ± 5.2
Visual search									
Center of Fixation [°]		5.5 ± 0.7	4.8 ± 0.7	4.4 ± 0.7	4.5 ± 0.8	1.4 ± 0.2	0.2 ± 0.4	0.4 ± 0.3	-0.2 ± 0.4
First orienting leftward [%]		7.3 ± 1.8	12.2 ± 2.5	25.8 ± 4.4	26.5 ± 4.5	34.1 ± 3.9	44.4 ± 4.3	58.7 ± 4.2	66 ± 3.9
Omission rate [%]	OL	72.1 ± 8.8	56.4 ± 9.4	74 ± 7.8	69.7 ± 9.4	2.5 ± 1.7	0 ± 0	2.5 ± 1.7	2.5 ± 1.7
	CL	42.7 ± 9.8	30.9 ± 8.9	45.1 ± 10.5	39.7 ± 9.9	3.8 ± 2	1.3 ± 1.3	1.3 ± 1.3	1.3 ± 1.3
	CR	19.6 ± 7.1	14.8 ± 6	17.2 ± 5.7	18.1 ± 5.6	0 ± 0	0 ± 0	1.3 ± 1.3	1.3 ± 1.3
	OR	4.4 ± 3.2	6.9 ± 4.2	25 ± 7.6	26.5 ± 9.1	1.3 ± 1.3	0 ± 0	0 ± 0	0 ± 0
Reaction time [s]	OL	10.5 ± 0.5	9.6 ± 0.7	10.7 ± 0.5	10.5 ± 0.6	2.9 ± 0.3	2.1 ± 0.1	2.8 ± 0.3	2.2 ± 0.2
	CL	7.9 ± 0.8	7.5 ± 0.7	8.4 ± 0.8	7.7 ± 0.8	2.9 ± 0.3	1.9 ± 0.2	2.4 ± 0.3	2.3 ± 0.2
	CR	5 ± 0.7	4.5 ± 0.7	6.1 ± 0.7	6.2 ± 0.5	1.7 ± 0.2	2.1 ± 0.2	2.5 ± 0.2	3.1 ± 0.3
	OR	3.5 ± 0.4	3.5 ± 0.5	6.8 ± 0.7	6.3 ± 0.8	2.3 ± 0.2	2.1 ± 0.2	2.5 ± 0.2	2.8 ± 0.2

Data are reported as Mean ± SEM. *OL* outer left, *CL* center left, *CR* center right, *OR* outer right referring to the target columns

#### Center of fixation

**Fig. 3:**
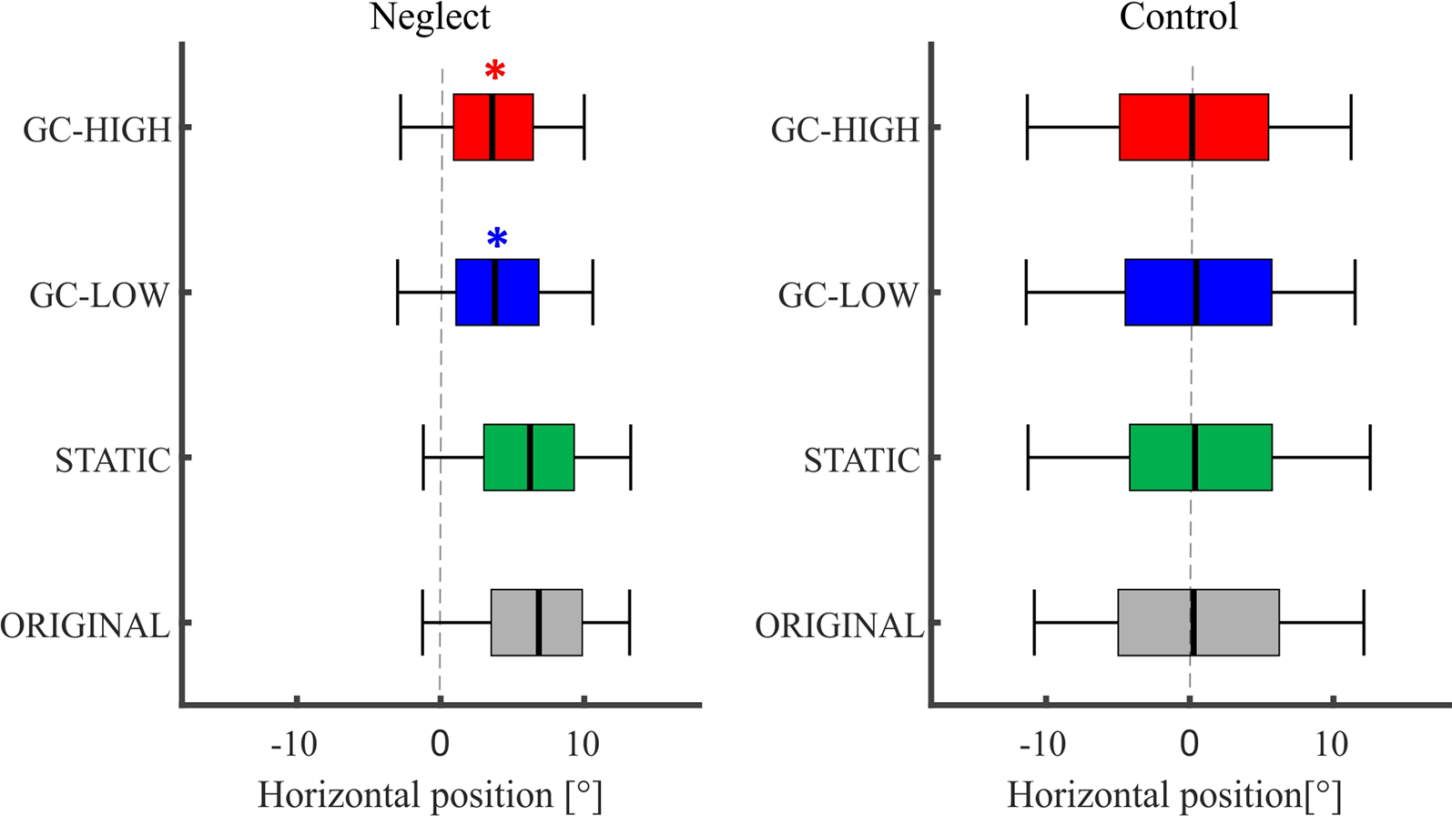
Center of fixation in the free viewing task. The distribution of fixations along the screen’s horizontal axis is depicted as a boxplot diagram, separately for neglect patients and controls as well as for the four different types of modification. The black median band represents the CoF, i.e. 50% of the fixations landed left and 50% right of this position. The boxes span the area from the lower quartile (left end) to the upper quartile (right end) and only 2.5% of all fixations were located outside the whiskers. In neglect patients, the pathological rightward shift of the CoF was significantly reduced under GC-HIGH and GC-LOW as compared to the ORIGINAL version of the picture (*p < 0.05). Further significant between-modification differences are reported in the main text

The CoF in the ORIGINAL condition in the FV task was 6.8° ± 0.8 in the neglect group and 0.3° ± 0.2 in the control group (Fig. 3, Table 2).

The ANOVA revealed a significant main effect of MODIFICATION (*F* (3, 36) = 24.12, *p* < 0.001), of GROUP (*F* (1, 38) = 32.51, *p* < 0.001), and a significant interaction MODIFICATION * GROUP (*F* (3, 36) = 14.21, *p* < 0.001).

The patients’ CoF under ORIGINAL was further right than the controls’ (*d* = 6.6 ± 0.8, *p* < 0.001). Furthermore, this pathological rightward shift of the patients’ CoF was significantly reduced by both GC modifications (GC-LOW: *d* = -3 ± 0.4; GC-HIGH: *d* = -3.2 ± 0.5, both *p* < 0.00)1, which also differed from STATIC (GC-LOW: *d* = -2.4 ± 0.3; GC-HIGH: *d* = -2.6 ± 0.4, both *p* < 0.001). GC modifications, however, could not completely normalize this bias, as the patients’ CoF under GC-HIGH was still significantly further right than the controls’ CoF under ORIGINAL (*d* = 3.3 ± 0.9, *p* = 0.001).

In the control group, the CoF did not differ between the different modifications (always *p* ≥ 0.846).

#### First orienting

**Fig. 4:**
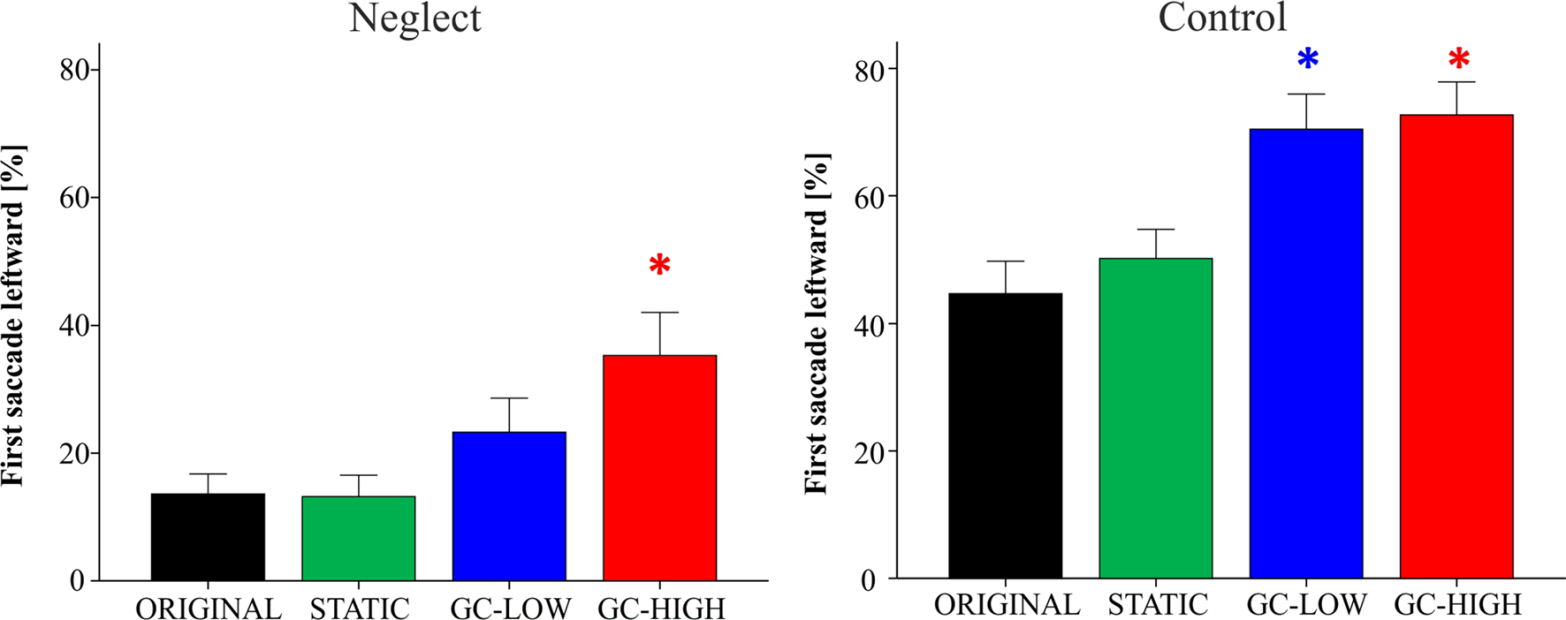
First orienting in the free viewing task. The first orienting is depicted as the percentage of trials in which the first saccade was directed leftward (i.e. into the neglected hemifield in the patients), separately for the neglect and the control group and the four modification conditions. Under GC-HIGH, neglect patients started their visual exploration more often on the left side of the screen (compared to ORIGINAL, *p < 0.05). The GC modifications also made the controls start more frequently on the left than under ORIGINAL. Further significant between-modification differences are reported in the main text. Error bars show the SEM

In the ORIGINAL condition the first saccade was directed leftward in 13.6% ± 3.1 of the FV trials in neglect patients versus 44.6% ± 5.1 in healthy controls (see also Table 2, Fig. 4).

The ANOVA for the First orienting in the FV task revealed a significant main effect of MODIFICATION (*F* (3, 36) = 32.67, *p* < 0.001) and of GROUP (*F* (1, 38) = 39.4, *p* < 0.001). The interaction MODIFICATION * GROUP was not significant (*F* (3,36) = 2.08, *p* = 0.12).

In the ORIGINAL condition, patients started their visual exploration significantly less often with a leftward saccade than the controls (*d* = − 31 ± 6.1, *p* < 0.001).

Within the neglect group, the percentage of leftward first saccades was higher in GC-HIGH than in all three other modifications (ORIGINAL: *d* = 21.7 ± 4.8, *p* < 0.001; STATIC: *d* = 22.1 ± 4.6, *p* < 0.001; GC-LOW: *d* = 12 ± 3.9, *p* < 0.022). The GC-HIGH modification almost led to a normalization of the patients’ first orienting, as there was no significant difference from the controls’ ORIGINAL condition (*d* = − 9.3 ± 8.3, *p* = 0.269).

Within the control group, the first saccade was more often directed leftwards in both GC modifications when compared to the ORIGINAL (GC-LOW: *d* = 25.8 ± 4.7; GC-HIGH: *d* = 28.1 ± 4.6, both *p* < 0.001) and to the STATIC (GC-LOW: *d* = 20.3 ± 3.7; GC-HIGH: *d* = 22.5 ± 4.3, both *p* < 0.001).

### Outcomes in the VS task

All the control subjects completed four VS blocks, the patients absolved on average 3.2 (SD = 0.8) blocks.

#### Center of fixation

**Fig. 5:**
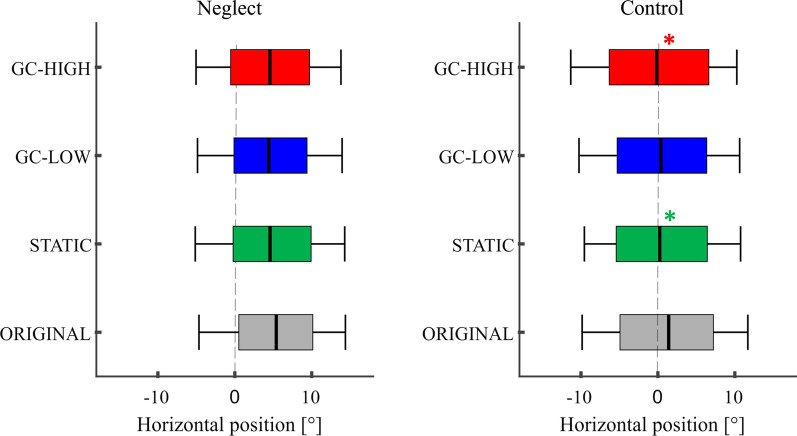
Center of fixation in de visual search task. The CoF is depicted as the black median band in this box plot diagram on the distribution of all fixations on the screen during the VS task (see Fig. 3 for further explanations). In control subjects, the CoF was significantly shifted to the left under STATIC and GC-HIGH as compared to the ORIGINAL version of the pictures (* *p* < 0.05). In the Neglect group, this shift was not significant

In the VS task, the CoF under ORIGINAL was 5.5° ± 0.7 in the neglect group and 1.4° ± 0.2 in the control group (see also Table 2, Fig. 5).

The ANOVA revealed a significant main effect of MODIFICATION (*F* (3, 34) = 8.81, *p* < 0.001) and of GROUP (*F* (1, 36) = 41.4, p < 0.001), but no significant interaction (*F* (3, 34) = 0.95, *p* = 0.427. Under ORIGINAL, the patients’ CoF was further right than the controls’ CoF (*d* = 3.1 ± 0.7, *p* ≤ 0.001).

Within the neglect group, there was no significant difference between the different modifications (all *p* ≥ 0.069).

In the control group, the CoF was significantly shifted further left in STATIC and GC-HIGH as compared to ORIGINAL (STATIC: *d* = − 1.2 ± 0.3, *p* = 0.002; GC-HIGH: *d* = − 1.6 ± 0.5, *p* = 0.016).

#### First orienting

**Fig. 6:**
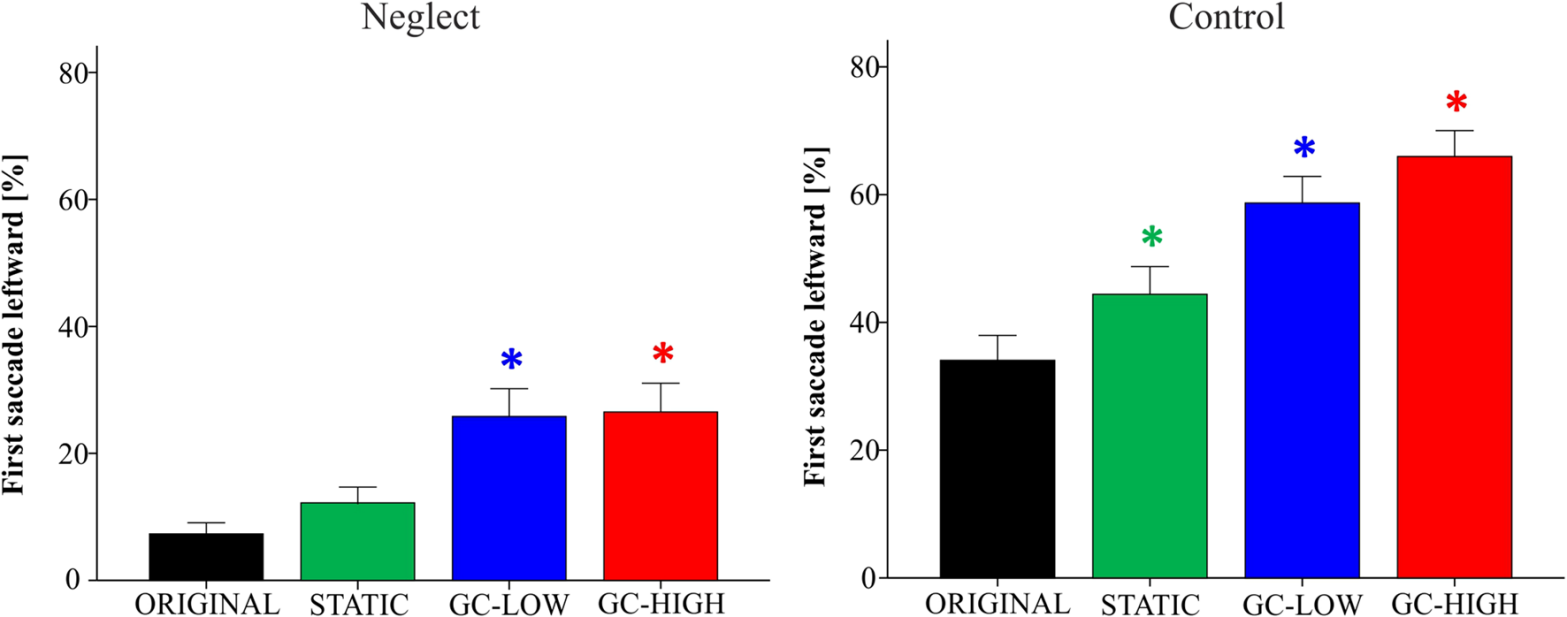
First orienting in the visual search task. Under the two GC-based modifications, neglect patients and control subjects started their visual search significantly more often on the left side of the screen (compared to ORIGINAL, * *p* < 0.05). The controls were also influenced by the STATIC mask, whereas its impact was not significant in neglect patients. Further significant between-modification differences are reported in the main text. Error bars show the SEM

In the ORIGINAL condition the first saccade was directed leftward in 7.3% ± 1.8 of the VS trials in neglect patients versus 34.1% ± 3.9 in healthy controls (Table 2, Fig. 6).

There was a significant main effect of MODIFICATION (*F* (3, 34) = 36.58, *p* < 0.001) and of GROUP (F (1, 36) = 51.87, p < 0.001) on the First orienting (percentage of leftward saccade). The interaction MODIFICATION * GROUP was not significant (*F* (3,34) = 2.26, *p* = 0.1).

Patients started less often on the left than the controls under ORIGINAL (*d* = − 26.8 ± 4.4, *p* < 0.001).

Within the neglect group, the percentage of first leftward saccades was significantly higher in both GC-modifications than in ORIGINAL (GC-LOW: *d* = 18.5 ± 4; GC-HIGH: *d* = 19.2 ± 3.5, both *p* < 0.001) and in STATIC (GC-LOW: *d* = 13.6 ± 3.8, *p* = 0.006; GC-HIGH: *d* = 14.3 ± 3.3, *p* = 0.001). Again, the GC-HIGH modification made that the patients’ first orienting approximated the performance of healthy controls under ORIGINAL (*d* = − 7.6 ± 5.9, *p* = 0.212).

In the control group, the two GC modifications but also the STATIC modification differed from the ORIGINAL (STATIC: *d* = 10.3 ± 2.5, *p* = 0.001; GC-LOW: 24.6 ± 3.6, *p* < 0.001; GC-HIGH: *d* = 31.9 ± 3.4, *p* < 0.001). Again, both GC modifications differed from STATIC (GC-LOW: *d* = 14.3 ± 3.6, *p* = 0.002; GC-HIGH: *d* = 21.6 ± 3.1, *p* < 0.001).

#### Omission rate

**Fig. 7:**
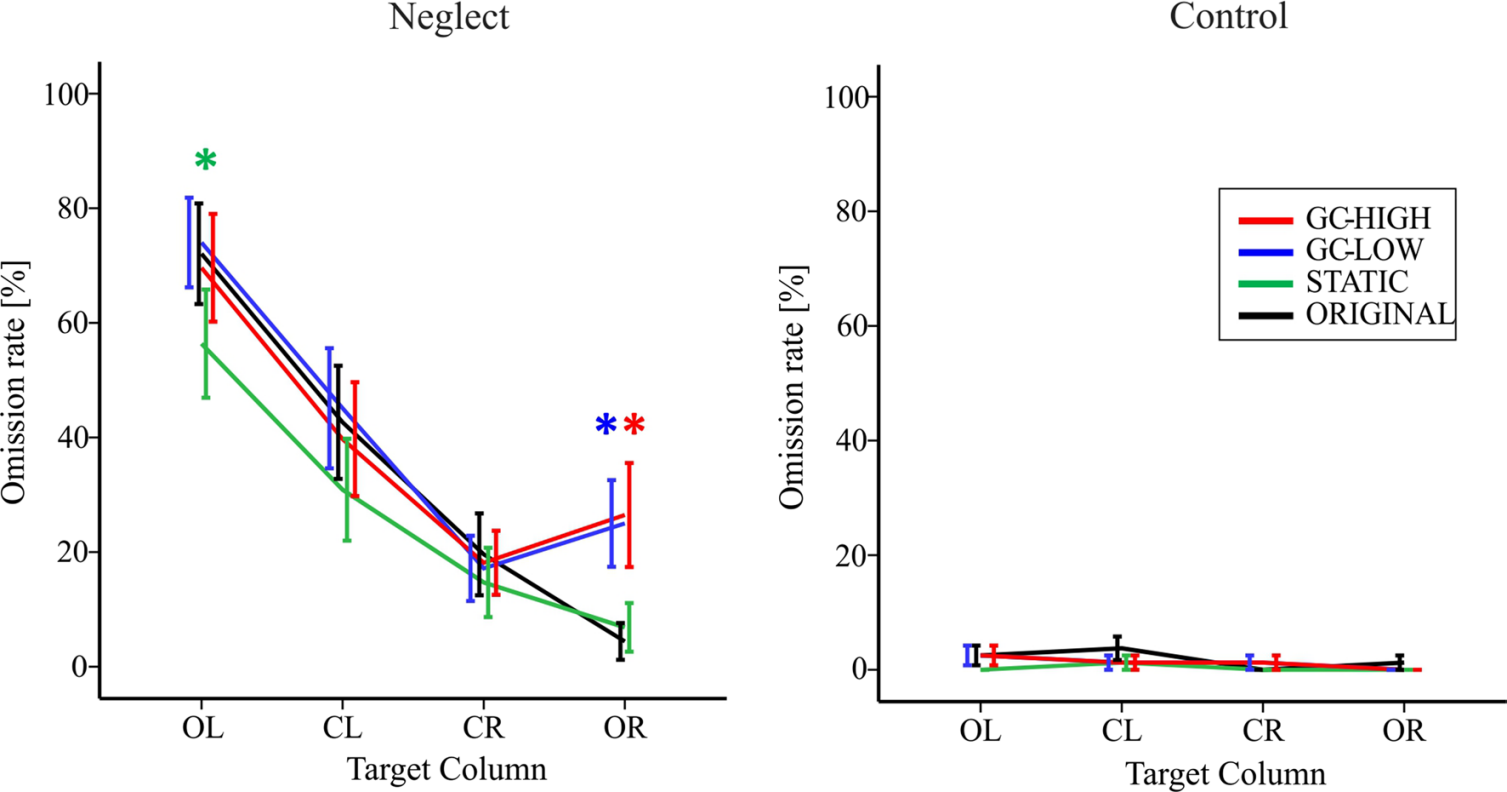
Target omissions in the VS task. The target omission rate [%] is shown separately for the two study groups and the four modification conditions (color-coded lines), in dependence of the four possible target positions (column: OL = outer left, CL = center left, CR = center right, OR = outer right) on the screen. Error bars show the SEM. The color-coded * represents a significant (*p* < 0.05) difference between the respective modification and the ORIGINAL

The neglect patients did not detect 34.7% ± 4.9 of the targets in ORIGINAL, whereas the control participants only missed 1.9% ± 0.7 on average (Table 2, Fig. 7).

The ANOVA revealed a significant main effect of MODIFICATION (*F* (3, 33) = 5.05, *p* = 0.005), of COLUMN (*F* (3, 33) = 31.79, *p* < 0.001) and of GROUP (*F* (1, 35) = 51.37, *p* < 0.001). Furthermore, there was a significant interaction of MODIFICATION * GROUP (*F* (3,33) = 3.57, *p* = 0.024) but no significant interaction of COLUMN * MODIFICATION * GROUP (*F* (9,27) = 1.31, *p* = 0.279).

For targets located in the outer left under ORIGINAL, patients revealed a greater rate of target omissions than controls (*d* = 69.6 ± 8.3, *p* < 0.001). For targets in the outer right under ORIGINAL, the omission rate did not statistically differ between groups (*d* = 3.2 ± 3.2, *p* = 0.337).

Within the neglect group, the omission rate in column OL was significantly higher than in column CL (*d* = 28.4 ± 3.8, *p* < 0.001), and in column CL higher than in CR (*d* = 22.2 ± 3.9, *p* < 0.001) and OR (*d* = 23.9 ± 5.9, *p* < 0.001). Regarding the impact of the MODIFICATION, patients had lower omission rates in STATIC than in the other three conditions (all *p* ≤ 0.014). A closer look at the differences between the modifications per column revealed that these differences were primarily driven by differences in the outer target columns (Fig. 7). Whereas there were no significant differences between the modifications in column CL and CR, in column OL the omission rate was smaller in STATIC than in the other three modifications (all *p* ≤ 0.022). In column OR, the omission rates were higher under both GC-modifications than under ORIGINAL or STATIC (all *p* ≤ 0.017).

In the control group the omission rates per column and modification were quite low ranging between 0 and 3.8%. There were no significant differences between target columns or modifications.

#### Reaction time

**Fig. 8:**
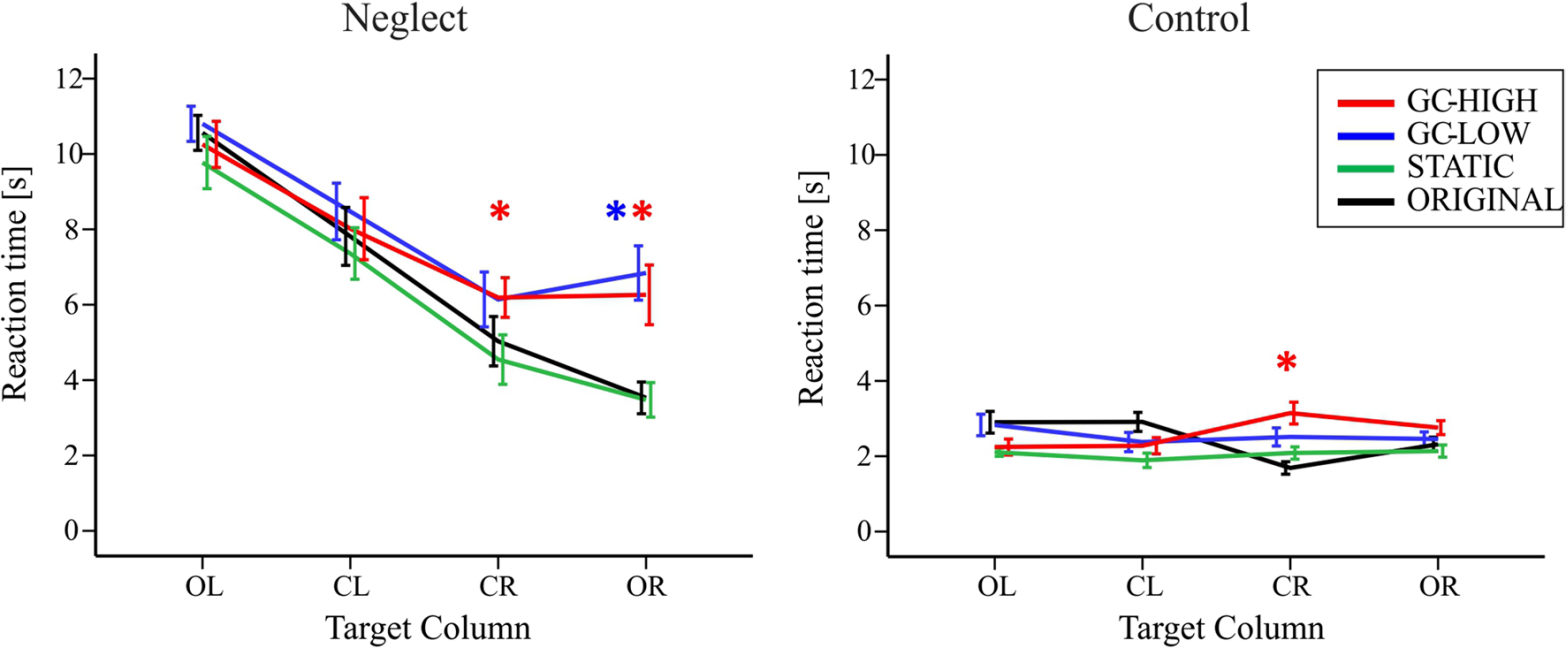
Reaction times in the VS task. Reaction time [s] until target detection is shown separately for the two study groups and the four modification conditions (color-coded lines) in dependence of the target location on the screen (column OL = outer left, CL = center left, CR = center right, OR = outer right). The color-coded * represents a significant (*p* < 0.05) difference between the respective modification and the ORIGINAL. Error bars show the SEM

The mean RT in ORIGINAL was 6.6 s ± 0.3 for the neglect and 2.5 s ± 0.1 for the control group (Table 2, Fig. 8).

The ANOVA revealed a significant main effect of MODIFICATION (*F* (3, 33) = 23.86, *p* < 0.001), of COLUMN (*F* (3, 33) = 57.14, *p* < 0.001) and of GROUP (*F* (1, 35) = 109.84, *p* < 0.001). The interactions of MODIFICATION * GROUP (*F* (3,33) = 7.35, *p* = 0.001) and COLUMN * MODIFICATION * GROUP (*F* (9,27) = 4.3, *p* = 0.002) were significant.

For targets in the outer columns (OL or OR) under ORIGINAL, patients yielded higher RTs than the controls (OL: *d* = 7.6 ± 0.5, *p* < 0.001; OR: *d* = 1.2 ± 0.4, *p* = 0.009).

Regarding the impact of target position, within the neglect group RTs were higher for targets located in OL than in CL (*d* = 2.4 ± 0.3, *p* < 0.001) and also higher in CL than in CR (*d* = 2.4 ± 0.2, *p* < 0.001) and OR (*d* = 2.9 ± 0.5, *p* < 0.001).

Regarding MODIFICATION, the RTs of neglect patients were significantly higher in both GC modifications than in ORIGINAL (GC-LOW: *d* = 1.3 ± 0.2; GC-HIGH: *d* = 1 ± 0.2, both *p* ≤ 0.001) and in STATIC (GC-LOW: *d* = 1.7 ± 0.2; GC-HIGH: *d* = 1.4 ± 0.2, both *p* < 0.001). Figure 8 shows that this difference was predominantly driven by significant differences in CR and OR. For targets located on the right, patients revealed higher RTs under the two GC-modifications than under STATIC (CR and OR: all *p* ≤ 0.001) and under ORIGINAL (GC-HIGH: CR, d = 1.2 ± 0.4, *p* = 0.048; OR, *d* = 2.7 ± 0.5, *p* < 0.001; GC-LOW: OR, *d* = 3.3 ± 0.5, *p* < 0.001).

Within the control group, reactions were slower under GC-HIGH than under STATIC (*d* = 0.6 ± 0.2, *p* = 0.015). A closer look at the impact of the target location revealed that this was predominantly driven by differences in column CR, where the RT was significantly higher under GC-HIGH than under STATIC (*d* = 1.1 ± 0.3, *p* = 0.027) and also under ORIGINAL (*d* = 1.5 ± 0.4, *p* = 0.003).

#### Single subject analyses of potential responders

We finally looked for potential individual responders among the patients by analyzing on a descriptive single subject basis the impact of the strongest modification (GC-HIGH) on the behavioral performance (omission rate, RT) in the VS task (see Additional file 5: Tables S5 and S6). An individual was defined as a responder if there was a ‘net benefit’ under the modification versus ORIGINAL, i.e. an improvement of the omission rate and/or RT for targets on the left (OL + CL) that was greater than a potential worsening on the right (OR + CR). With respect to target omissions, only four (patient ID 13, 19, 20, 22) out of 19 patients improved under the modification (Additional file 5: Table S5). Regarding RTs, we identified three (patient ID 5, 13, 18) out of 19 patients who yielded such a net benefit under the modification (Additional file 5: Table S6). Taken together, in these descriptive analyses we identified six out of 19 patients who might have benefitted from the GC modification regarding performance in the VS task. When looking at the patient characteristics (age, time since lesion, neglect severity) in this small sample, we could not determine any specific predisposing features, in which these ‘responders’ differed from the rest of the group.

### Patients’ report

The patients subjectively rated both the FV task (8.7, SD = 1.6) and the VS task (8.2, SD = 1.9) as highly pleasant. Only 6 of 19 patients reported any notice of the modifications at all, e.g. by stating “image was sometimes less clear” or “seemed as if I’d need glasses”.

## Discussion

This study investigated whether a GCD-based lateralized modification of a scene’s visual salience can counteract the imbalanced attentional priority map and positively influence (or even normalize) the visual exploration behavior of patients with left hemispatial neglect. We applied different static and dynamic (GC) masks that permanently reduced the contrast/color saturation of objects in the ipsilesional hemispace. Thereby we relatively increased the salience and attentional weight of objects in the neglected contralesional hemispace. To assess the effectivity of our modifications, we measured (i) the median horizontal position of all fixations in the scene (CoF) as a marker of the egocentric spatial attention bias, (ii) the direction of first orienting (percentage of first saccades going leftward) as a marker of the very early (“pre-attentive”) spatial bias and (iii) the performance when searching for targets in a visual scene (omission rate and RT) as an indicator of the functional impairment during an everyday activity. We used highly accurate eye tracking and fast algorithms to successfully implement the GCD modification and to directly measure the effects during two different visuo-motor tasks (FV and VS), which are known to have high sensitivity and high test–retest reliability in the assessment of spatial neglect [20, 23, 41, 42]. A group of healthy subjects constituted the control to prove if a normalization of the patients’ exploration behavior was achieved and to ensure that any findings in the patients were not merely due to the basic composition of the stimulus images.

The main results were the following: Without modification, patients with left hemispatial neglect exhibited the typical ipsilesional attention bias in terms of a strong rightward deviation of their CoF. This was evident in both task conditions, especially in FV but also during VS, which is in line with previous reports that showed that hemispatial neglect affects especially the bottom-up stimulus-driven exploration but also the goal-driven VS under top-down control [16, 20, 23, 48]. This bias was already present in the very early phase of orienting, because less than 15% of all first saccades in FV and 8% in VS went towards the (neglected) left hemifield, as compared to the controls’ 45% and 34%, respectively. During VS, we also observed the neglect-typical severe impairment in detecting targets in the contralesional hemifield, i.e. an increase in total omissions and in reaction times for left-side targets. Our modifications had a significant impact on the patients’ exploration pattern. Resembling a typical dose effect, we observed the greatest effects for the strongest modification (GC-HIGH). This combined static-dynamic modification (i) almost halved the degree of the patients’ ipsilesional attention bias during FV and (ii) increased the likelihood to start the visual exploration in the neglected left hemifield by about 20% in both FV and VS. The modification could, however, (iii) not improve the detection of targets located in the left half of the scene corresponding to the neglected hemifield and even worsened the detection of targets in the right part of the scene. To specify, the GC-modifications led to an increase of total omissions and RTs for targets located in the right (ipsilesional) hemispace. While the RT increase for targets in the outmost right part of the scene could partly be explained by a total unawareness and omission of the target (because this was automatically counted as the maximum RT), the RT increase for targets located in the scene’s part right of the center was due to a real delay in responding to the target. The reason for both effects may have been the blur induced by the GC-mask, which led to a reduced visibility/salience of right-side targets.

Additional single subject analyses revealed that six out of the 19 patients may still have benefitted from the strongest GC modification in their VS performance. These potential ‘responders’ showed an individual improvement of the omission and/or RT for left-side targets that was not offset by any blur-related worsening on the right. In this small patient sample, however, we could not identify individual characteristics (e.g., neglect severity) that could be taken as favorable prognostic factors for responding to a GC-based intervention.

Taken together, our GCD-based modification was partly effective in patients with hemispatial neglect, as it reduced (though not normalized) the ipsilesional fixation bias that is commonly taken as an indicator of the egocentric spatial attention bias in neglect [22, 23, 48]. Although the GCD-based intervention was shown to be feasible and well tolerated by the patients (some found it even pleasant) and partly beneficial for the patients’ visual exploration behavior, a potential application as a rehabilitative tool would certainly require further testing of its effectiveness. This would include the translation of effects to an improvement of everyday activities and a persistence of effects in the long term.

Unfortunately, the alleviation of the ipsilesional fixation bias in our approach did not yet go along with a functionally relevant improvement in finding targets in the neglected hemifield (except for a few potential individual responders, see above). This discrepancy somehow questions the assumption that the CoF is equivalent to the center of attention and that the fixation bias is equal to the general attention bias. A similar dissociation of oculomotor performance and attentional processing was found in a previous study, in which patients with ‘remitted’ neglect at the chronic stage revealed increased visual exploration of the left hemifield but without a concomitant improvement of the reaction time for targets located there [24]. It may, however, also indicate that our chosen modification was too weak to completely normalize the patients’ attention bias and to significantly improve search performance. However, from a practical point of view, the detection of right-side targets under the mask was already hampered and a stronger mask would possibly provoke more harm on the right than benefit on the left. Another limitation of our approach is that the degree of the ipsilesional attention bias together with clinical neglect severity varies across patients [48]. Hence, it is difficult to counterbalance a different individual tilt of the attentional priority map by a one-fits-all modification mask. This may be one reason, why in our study not the whole group but only a few individual patients improved their search performance under the influence of the GC mask. Aiming at a more individualized treatment (“precision medicine”), Bays and colleagues [16] previously used a computerized algorithm that increased or decreased the strength of the salience modification in the right hemifield on a rapid trial-by-trial base (not GCD) to finally find the optimal counterbalance for the individual spatial attention bias of each patient with left neglect. Although they were quite successful in almost normalizing the patients’ attention bias, the improved performance for target detection in the neglected left hemifield was also at the cost of a worse performance for right-side targets under the mask.

The question remains whether GCD-based approaches of salience modification could still be suitable tools for neglect rehabilitation in the future. Given the further technological evolution of see-through head-mounted displays [50] or computerized glasses as well as integrated remote eye-trackers, the good tolerance and reported pleasance of such kind of intervention by the patients certainly makes it a feasible and worthwhile approach. Furthermore, especially during FV, the GCD-based modification of visual salience in the ipsilesional hemispace could successfully induce a relative increase of salience/attractivity of objects in the contralesional hemifield and a shift of exploratory eye movements towards contralesional hemispace in our patients. While there was no clear benefit during a goal-driven VS task in our study on the group level, we cannot exclude that such an induced shift of exploratory eye movements (reflecting overt shifts of attention) may still be beneficial for individual neglect patients in their everyday life.

## Conclusions

GCD technology modifying the input/salience of the visual surrounding can positively influence visual exploration (attentional shifts) in hemispatial neglect patients. While an alleviation of the neglect-related ipsilesional fixation bias can be achieved by GCD-based modifications, a concomitant functionally relevant improvement of spatial attention (e.g., better detection of contralesional targets) remains to be proven. Further research is necessary to investigate the influence of individually customized GC-based modifications depending on the patient’s individual degree of the ipsilesional attention bias and neglect severity. Furthermore, to allow conclusions about the functional relevance and ecological validity, GCD-based modifications during augmented reality via see-through head-mounted displays should be tested in patients with hemispatial neglect during activities of daily living. If beneficial and persistent effects were induced, GCD-based modifications could represent a promising approach for an individualized rehabilitative neglect treatment in the future.

## Supplementary Information

**Additional file 1. **Scanpath of one exemplary patient (red square = current gaze point) on a stimulus image in its original form (task: free viewing).

**Additional file 2. **Scanpath of the same patient on an image under the STATIC modification.

**Additional file 3. **Scanpath of the same patient on an image under the GC-LOW modification.

**Additional file 4. **Scanpath of the same patient on an image under the GC-HIGH modification.

**Additional file 5. **Additional tables.

## Data Availability

The data that support the conclusions of this manuscript and the Matlab script which was used to program the stimulus modification are available from the corresponding author upon reasonable request.
