# Supplementary material for: Gaze-contingent display technology can help to reduce the ipsilesional attention bias in hemispatial neglect following stroke
Source: J Neuroeng Rehabil. 2022 Nov 16;19:125. doi: 10.1186/s12984-022-01104-5 (PMC9670469; doi:10.1186/s12984-022-01104-5)
Supplement: Supplementary file 5 — Additional file 5. Additional tables. [file 12984_2022_1104_MOESM5_ESM.pdf]

Supplemental Material

‘Gaze-contingent display technology can help to reduce the ipsilesional attention bias in hemispatial neglect following stroke’ by

Lisa Kunkel genannt Bode, Anna Sophie Schulte, Björn Hauptmann, Thomas F. Münte, Andreas Sprenger, Björn Machner

Table S.1: Individual demographic, clinical and neuropsychological characteristics of the patients with spatial neglect

ID	Age	Sex	Type of stroke (hemorrhagic, ischemic)	Lesion location (right hemisphere)	Time since stroke [days]	Barthel Index	Text	Word list	Bells: CoC	Line Bisection	Figure copying (Ogden)	Clock	Rating of convenience (1-10)	
													FV	VS
**1**	75	m	h	Thalamus	118	45	2	2	0.23	8	4	5	10	10
**2**	61	f	i	Art. cerebri media	18	55	3	3	0.08	6	0	5	7	5
**4**	78	f	h	Right frontal lobe	39	40	0	1	0.23	6	2	5	9	5
**5**	58	m	h	Basal ganglia	247	50	NaN	NaN	0.03	42	2	NaN	10	10
**6**	79	m	h	Basal ganglia	58	55	2	0	0.15	8	0	4	10	8
**7**	67	f	i	Art. cerebri posterior	45	45	42	11	0.1	52	4	4	10	10
**8**	55	m	i	Art. cerebri media	30	30	1	0	0.09	13	3	5	8	8
**9**	58	f	h	Basal ganglia	96	40	41	2	0.92	85	4	2	10	10
**10**	52	f	i	Art. choroidea anterorior	91	30	34	11	0.93	88	4	4	8	8
**11**	66	f	i	Art. cerebri media	15	40	3	5	0.07	NaN	3	2	9	9
**12**	79	f	h	Parietal lobe	97	45	1	2	0.97	30	4	4	9	9
**13**	58	m	i	Art. cerebri media	39	45	0	1	0.13	11	1	NaN	5	5
**14**	79	m	i	Art. cerebri media	36	55	21	2	0.66	36	4	4	10	10
**16**	79	f	h	Right hemisphere	53	55	8	5	0.21	NaN	4	5	8	8
**17**	58	m	i	Art. cerebri media and Art. cerebri posterior	52	35	112	12	0.91	55	4	5	8	8
**18**	75	m	i	Art. cerebri media	80	30	31	5	0.44	26	3	2	10	9
**19**	57	m	i	Art. cerebri media	530	30	22	0	0.66	25	3	4	10	10
**20**	60	m	i	Art. cerebri media	68	35	3	2	0.02	9	4	2	5	5
**22**	66	f	i	Art. cerebri media	276	45	6	3	0.49	61	3	3	NaN	NaN

Table S.2: Individual characteristics of the healthy control participants

ID	Age	Sex	Bells: CoC	Line bisection: Deviation in mm	Rating of convenience (1 – 10)	
					FV	VS
**1**	35	f	0	-1	8	10
**2**	57	f	0	-1	7	8
**3**	55	m	0	-1	5	10
**4**	48	f	0	0	7	8
**5**	34	f	0	-3	3	9
**6**	62	f	0	-3	6	8
**7**	65	f	0	-1	8	6
**8**	60	m	0.002	0	3	9
**9**	46	f	0.029	2	8	9
**10**	56	f	0	2	3	7
**11**	66	f	-0.004	10	10	10
**12**	48	f	0	1	6	8
**13**	57	f	0	2	8	7
**14**	52	m	0	-1	7	8
**15**	57	f	0	-3	8	8
**16**	66	f	0	-1	9	9
**17**	41	m	0	2	8	9
**18**	74	f	0	-4	10	9
**19**	59	m	0.004	2	10	10
**20**	52	m	0	-1	10	9
**21**	59	m	-0.012	-2	5	8
**22**	55	f	0	-2	5	7

Table S.3: Individual performance of neglect patients during free viewing under the different modification conditions

Center of Fixation [°]				
**ID**	**ORIGINAL**	**STATIC**	**GC-LOW**	**GC-HIGH**
**1**	4.95	4.08	0.83	0.55
**2**	3.43	2.21	0.15	-1.37
**4**	5.88	1.62	0.9	1.68
**5**	3.84	4.57	2.16	2.67
**6**	11	10.58	7.66	8.69
**7**	6.37	5.66	2.2	0.91
**8**	4.09	4.54	0.66	-0.52
**9**	8.94	8.09	8.55	7.16
**10**	10.65	10.31	8.65	9.03
**11**	0.71	0.52	-2.05	-3.16
**12**	9.43	10.15	7.93	7.43
**13**	0.5	1.08	-1.69	-1.67
**14**	11.59	12.07	9.01	8.03
**16**	7.26	4.6	2.19	2.58
**17**	7.32	10.26	7.88	7.76
**18**	5.25	2.74	-1.31	-0.08
**19**	10.08	9.51	7.55	9.02
**20**	9.42	7.93	5.15	3.39
**22**	9.21	7.86	5.6	6.34

First Orienting leftward [%]				
**ID**	**ORIGINAL**	**STATIC**	**GC-LOW**	**GC-HIGH**
**1**	5	5	5	30
**2**	20	20	50	100
**4**	38	60	67	70
**5**	13	14	13	20
**6**	14	8	20	7
**7**	0	12	0	13
**8**	0	20	60	40
**9**	0	13	8	13
**10**	14	13	20	13
**11**	40	20	47	73
**12**	38	0	0	33
**13**	20	33	67	93
**14**	0	0	5	15
**16**	27	7	27	27
**17**	0	0	0	50
**18**	14	0	20	36
**19**	0	0	0	0
**20**	11	20	24	21
**22**	5	5	11	17

Table S.4: Individual performance of neglect patients during visual search under the different modification conditions

Center of Fixation [°]				
**ID**	**ORIGINAL**	**STATIC**	**GC-LOW**	**GC-HIGH**
**2**	2.35	0.56	1.38	2
**4**	5.98	5.72	2.56	2.3
**5**	2.62	2.21	1.31	-0.22
**6**	4.93	5.7	4.1	5.19
**7**	5.17	4.09	5.2	5.95
**8**	4.51	3.88	2.34	2.05
**9**	8.33	9.37	8.45	9.89
**10**	8.47	8.82	7.53	10.2
**11**	1.63	1.28	-0.17	-0.13
**12**	10.22	9.54	8.97	8.98
**13**	1.85	1.55	2.26	2.95
**14**	7.05	6.3	8	8.01
**16**	-0.22	1.47	2.93	4.03
**17**	6.54	5.76	5.31	4.31
**18**	8.05	4.15	-0.33	-0.92
**19**	7.42	7.09	5.9	5.83
**20**	6.95	4.41	7.18	5.93
**22**	7.87	4.46	6.02	5.28

First Orienting leftward [%]				
**ID**	**ORIGINAL**	**STATIC**	**GC-LOW**	**GC-HIGH**
**2**	25	19	38	57
**4**	15	30	33	57
**5**	11	11	40	30
**6**	5	21	40	11
**7**	5	5	30	35
**8**	10	11	50	40
**9**	14	13	7	14
**10**	0	0	0	18
**11**	0	7	47	33
**12**	10	10	10	10
**13**	15	30	60	60
**14**	0	0	5	5
**16**	14	20	27	40
**17**	0	30	22	13
**18**	7	7	29	29
**19**	0	0	0	0
**20**	0	0	0	0
**22**	0	5	26	25

Table S.5: Neglect patients' individual target omission rates [%] under the ORIGINAL versus the GC-HIGH condition in the visual search task in dependence of the target location (column)

ID	Col	ORIGINAL	GC_HIGH	Difference	ID	Col	ORIGINAL	GC_HIGH	Difference
**2**	OL	33	0	-33	**12**	OL	100	100	0
	CL	0	0	0		CL	100	100	0
	CR	0	33	33		CR	50	0	-50
	OR	0	0	0		OR	0	0	0
**4**	OL	100	0	-100	**13**	OL	50	0	-50
	CL	0	100	100		CL	0	0	0
	CR	0	0	0		CR	0	0	0
	OR	0	100	100		OR	0	0	0
**5**	OL	100	100	0	**14**	OL	100	100	0
	CL	50	0	-50		CL	75	50	-25
	CR	0	25	25		CR	25	25	0
	OR	50	75	25		OR	0	25	25
**6**	OL	33	100	67	**16**	OL	0	0	0
	CL	0	50	50		CL	33	0	-33
	CR	0	25	25		CR	33	33	0
	OR	0	50	50		OR	0	33	33
**7**	OL	100	100	0	**17**	OL	100	100	0
	CL	0	50	50		CL	50	100	50
	CR	0	0	0		CR	0	0	0
	OR	25	0	-25		OR	0	100	100
**8**	OL	0	50	50	**18**	OL	100	67	-33
	CL	0	0	0		CL	67	0	-67
	CR	0	0	0		CR	33	33	0
	OR	0	0	0		OR	0	100	100
**9**	OL	100	100	0	**19**	OL	100	100	0
	CL	100	100	0		CL	100	67	-33
	CR	67	67	0		CR	0	0	0
	OR	0	0	0		OR	0	0	0
**10**	OL	100	100	0	**20**	OL	67	50	-17
	CL	100	100	0		CL	25	33	8
	CR	100	67	-33		CR	0	0	0
	OR	0	67	67		OR	0	0	0
**11**	OL	67	67	0	**22**	OL	75	50	-25
	CL	0	0	0		CL	25	25	0
	CR	0	0	0		CR	25	0	-25
	OR	0	0	0		OR	0	0	0

Col: One of four columns (OL outer left, CL center left, CR center right and OR outer right), in which the target could be located on the screen.
Individual "responders" to the GC-HIGH modification are highlighted in color. An individual was defined as a responder if there was a net benefit under the modification, i.e. the difference/change GC-HIGH – ORIGINAL fulfilled the following criteria: OL + CL < 0 and either a) OR + CR ≤ 0 or b) 0 < OR + CR < |OL + CL|.

Table S.6: Neglect patients' individual reaction times [s] under the ORIGINAL versus the GC-HIGH condition in the visual search task

ID	Col	ORIGINAL	GC_HIGH	Difference	ID	Col	ORIGINAL	GC_HIGH	Difference
2	OL	8.66	11.94	3.28	12	OL	12	12	0
	CL	2.5	3.54	1.05		CL	12	12	0
	CR	4.61	6.19	1.58		CR	7.28	7.54	0.26
	OR	2.09	3.35	1.26		OR	1.99	5.18	3.19
4	OL	12	5.88	-6.13	13	OL	7.95	3.8	-4.15
	CL	6.3	12	5.72		CL	3.17	3.48	0.31
	CR	6.78	7.88	1.09		CR	2.25	3.14	0.89
	OR	2.53	12	9.48		OR	2.44	2.39	-0.05
5	OL	12	12	0	14	OL	12	12	0
	CL	7.19	3.39	-3.8		CL	10.16	9.65	-0.50
	CR	4.15	4.57	0.42		CR	6.37	8.4	2.03
	OR	8.69	11.2	2.51		OR	3.82	7.02	3.20
6	OL	10.75	12	1.26	16	OL	7.80	6.47	-1.32
	CL	6.74	8.66	1.92		CL	6.50	4.26	-2.24
	CR	5.76	7.83	2.07		CR	5.12	8.49	3.37
	OR	3.47	7.89	4.42		OR	4.94	6.6	1.66
7	OL	12	12	0	17	OL	12	12	0
	CL	6.65	8.99	2.34		CL	10.33	12	1.68
	CR	5.62	4.94	-0.68		CR	2.67	6.41	3.74
	OR	4.76	3.72	-1.05		OR	3.99	12	8.01
8	OL	5.62	10.63	5.01	18	OL	12.00	9.23	-2.77
	CL	4.68	4.12	-0.56		CL	11.69	4.77	-6.92
	CR	2.98	5.76	2.78		CR	5.99	6.13	0.14
	OR	2.58	3.14	0.56		OR	4.66	12	7.34
9	OL	12	12	0	19	OL	12	12	0
	CL	12	12	0		CL	12	11.61	-0.39
	CR	9.07	9.34	0.27		CR	3.42	6.3	2.88
	OR	4.88	6.95	2.07		OR	2.23	5.72	3.49
10	OL	12	12	0	20	OL	9.02	7.8	-1.21
	CL	12	12	0		CL	4.84	6.21	1.37
	CR	12	8.73	-3.27		CR	1.10	2.05	0.95
	OR	3.24	9.15	5.91		OR	2.25	4.49	2.23
11	OL	10.06	11.43	1.36	22	OL	10.22	9.33	-0.89
	CL	4.99	6.15	1.16		CL	6.90	9.42	2.52
	CR	2.45	6.83	4.39		CR	4.68	2.57	-2.11
	OR	2.07	2.85	0.78		OR	1.87	2.81	0.94

Col: One of four columns (OL outer left, CL center left, CR center right and OR outer right), in which the target could be located on the screen.

Individual "responders" to the GC-HIGH modification are highlighted in color. An individual was defined as a responder if there was a net benefit under the modification, i.e. the difference/change GC-HIGH – ORIGINAL fulfilled the following criteria: $OL + CL < 0$ and either a) $OR + CR \leq 0$ or b) $0 < OR + CR < |OL + CL|$.
